# Analysis of trabecular bone microstructure in osteoporotic femoral heads in human patients: in vivo study using multidetector row computed tomography

**DOI:** 10.1186/s12891-015-0848-z

**Published:** 2016-01-12

**Authors:** Mitsuru Munemoto, Akira Kido, Yoshihiro Sakamoto, Kazuya Inoue, Kazuyuki Yokoi, Yasushi Shinohara, Yasuhito Tanaka

**Affiliations:** Department of Orthopaedic Surgery, Nara Medical University, 840 Shijocho, Kashihara, Nara 634-8521 Japan; Department of Orthopaedic Surgery, Okanami General Hospital, 1784 Uenokuuwamachi, Iga, Mie 518-0842 Japan

**Keywords:** Trabecular structure, Microstructure, Femoral head, Osteoporosis

## Abstract

**Background:**

Lag screw position is very important in the treatment of intertrochanteric femoral fracture to prevent complications such as screw cut-out. Current studies recommend central or inferior placement of the lag screw on the anteroposterior radiograph, and central placement on the lateral radiographs. These reports are based on radiographic evaluation, but few studies have investigated the importance of bone quality at the site of lag screw placement. In this study, we used multidetector row computed tomography (MDCT) to perform in vivo evaluation of the bone microstructure of the femoral head in patients with intertrochanteric femoral fractures.

**Methods:**

This study was approved by the Ethics Committee of Okanami General Hospital. MDCT images were obtained in our hospital from ten patients who had sustained intertrochanteric femoral fracture. Patients who needed computed tomography to confirm fracture morphology were included. We defined six areas as regions of interest (ROI): ROI 1–3 were defined as the femoral head apex area, and ROI 4–6 were defined as the femoral neck area. Trabecular microstructure parameters, including mean bone volume to total volume (BV/TV), trabecular thickness (Tb.Th), trabecular separation (Tb.Sp), and structure model index (SMI), were evaluated with bone analysis software (TRI/3D-BON). Statistical analyses were performed using EZR software; each parameter among the ROIs was statistically evaluated by analysis of variance (ANOVA) and Tukey’s test. Statistical significance was established at *p* < 0.05.

**Results:**

In the apical area, all parameters indicated that ROI 1 (superior) had the highest bone quality and ROI 2 (central) was higher in bone quality than ROI 3 (inferior). In the femoral neck, all parameters indicated that bone quality was significantly greater in ROI 6 (inferior) than ROI 5 (central).

**Discussion and Conclusions:**

We could evaluate bone quality with clinical MDCT in vivo. Bone quality in the central area of the femoral head apical was greater than in the inferior area, and bone quality in the inferior area of the femoral neck was greater than in the central area. Recognizing which area of femoral head has greater bone quality may lead to a better clinical result in treating intertrochanteric femoral fracture.

## Background

The Japanese population is aging faster than any other in the world, and one effect of this trend is that the incidence of proximal femoral fracture in Japan has rapidly increased. There were 148,000 proximal femoral fractures in Japan in 2007, which was 2.78 times higher than in 1987 [1]. After a proximal femoral fracture, patient quality of life decreases and morbidity and mortality increase [2]. Therefore, it is very important to treat these fractures appropriately. In Japan, osteosynthesis is used to treat intertrochanteric femoral fractures. One of the most serious complications in these treatments is cut-out of the lag screw [3]. To prevent this complication, appropriate reduction and screw positioning are important [4]. It has been suggested that the appropriate position for lag screw placement is in the central or inferior area on the anteroposterior (AP) X-ray view, and in the central region on the lateral view [3, 5–10]. Although there are several reports based on radiographic evaluation, few reports have investigated bone quality in the area of lag screw placement.

Previous studies have performed evaluation of trabecular bone microstructure with micro computed tomography (μCT), but only in ex vivo analyses [11–15]. Recently, there have been reports of in vivo microstructural analyses with multidetector row computed tomography (MDCT); these reports emphasized the usefulness of evaluating the bone microstructure with MDCT [16–19]. Ito et al. described the correlation between microstructure parameters and vertebral bone quality based on MDCT [16]. Baum et al. found that microstructure parameters most accurately predict absolute and relative femoral bone quality in cadaver study [17]. Sakamoto et al. reported a microstructural analysis of the humeral greater tuberosity in patients with rotator cuff tears [18]. Lu et al. showed that MDCT might have the potential to characterize the trabecular pattern and distribution of the proximal femur [19]. We therefore considered it important to use MDCT to evaluate the bone quality of the femoral head in the area of lag screw replacement.

In this study, we used MDCT images to investigate the trabecular bone microstructure of osteoporotic human femoral heads. Our aim was to evaluate the bone quality of the femoral head in the area recommended for the screw insertion based on trabecular bone microstructure in vivo.

## Patients and methods

This study was performed in accordance with the principles of the Declaration of Helsinki and was approved by the ethics committee at our institution. This study was a prospective case series that was approved by the Ethics Committee of Okanami General Hospital and we obtained written informed consent from participants. Between April 2012 and August 2012, we recruited ten consecutive patients who had sustained intertrochanteric fracture and required MDCT imaging. All patients were diagnosed by plain radiography, and MDCT was performed for preoperative evaluation of fracture type on the injured side; we used images of the uninjured side for this study.

The patients comprised two men and eight women with a median age of 85.1 years (range, 73–96 years). Patients with previous hip fracture or surgery, osteoarthritis of the hip, malignant tumor in any part of the body, or receiving bone modifying medication were excluded.

**Fig. 1:**
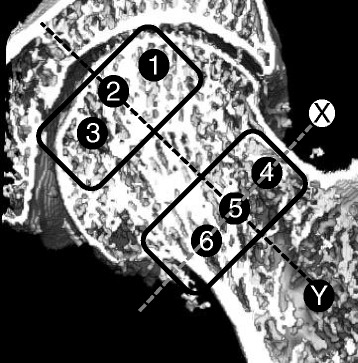
Regions of interest (ROI) in the femoral head. Line X: Femoral neck axis. Line Y: The line perpendicular to the neck axis in the femoral neck isthmus. ROI 1–3 were defined as the apical area. ROI 4–6 were defined as the neck area. ROI 1–3 were located on the extension of ROI 4–6, parallel to the femoral neck axis

Before surgery, an MDCT scan was performed with an Aquilion 64 CT scanner (Toshiba, Tokyo, Japan) using a standard protocol (120 kV, 250 mA, collimation of 0.5 mm, and reconstruction index of 0.3 mm) to evaluate bone quality. The scans were performed under the following conditions: field of view of 200 mm and pixel matrix of 512 × 512. For the morphometric analysis, specific regions of interest (ROIs) were defined within the femoral head (Fig. 1). All ROIs were located in the center of the lateral view. On the coronal view of the femoral head, ROI 4–6 were defined as the femoral neck area. Each area in the neck was on the line perpendicular to the neck axis in the femoral neck isthmus. ROI 5 was in the center of the neck, ROI 4 was in the superior neck, and ROI 6 was in the inferior neck. ROI 4 and 6 were positioned 5 mm deep to cortical bone. ROI 1–3 were defined as the femoral head apex area. ROI 1–3 were designed to be located as extensions of ROI 4–6, parallel to the femoral neck axis. Each area in the apex was 5 mm deep to subchondral bone. We defined ROI 1 as superior, ROI 2 as central, and ROI 3 as inferior. Each ROI had a cylindrical shape, with a diameter of 5 mm and a depth of 10 mm.

**Fig. 2:**
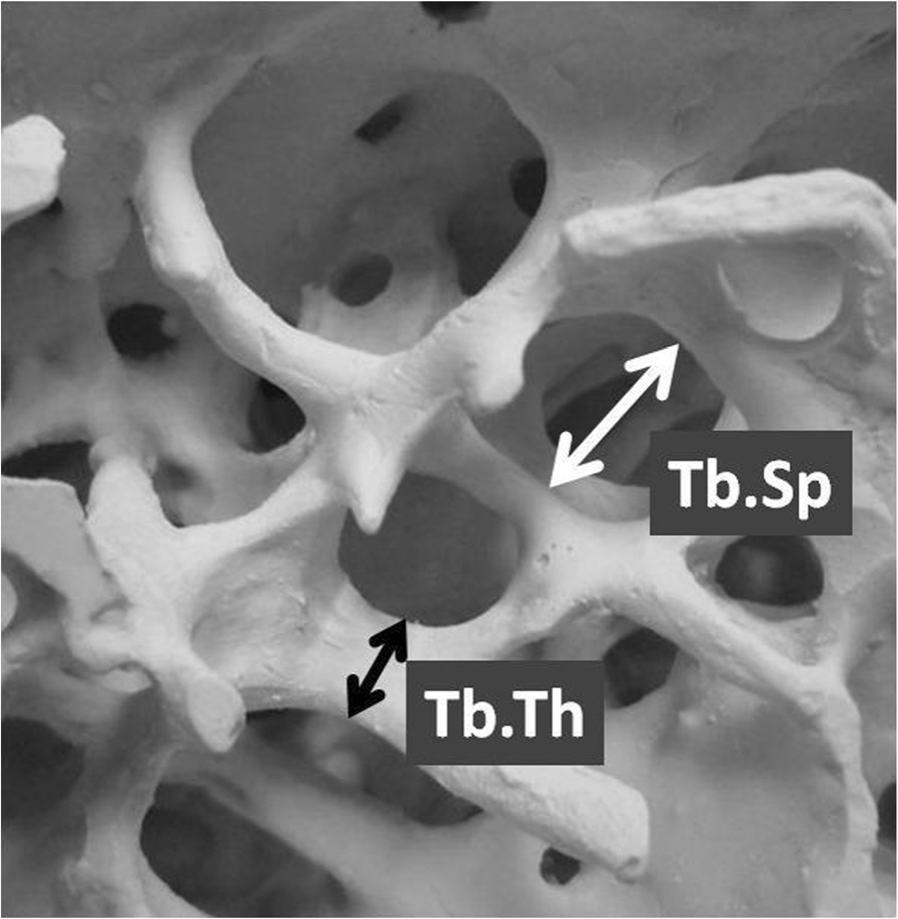
Trabecular microstructural parameters (reprinted from the literature with permission) [18]. The *black arrow* indicates the trabecular thickness (Tb.Th, μm), and the *white arrow* indicates the trabecular separation (Tb.Sp, μm)

After MDCT, imaging data were transferred to a workstation, and the trabecular microstructure parameters were measured using three-dimensional (3D) image analysis software (TRI/3D-BON; RATOC System Engineering Co., Tokyo, Japan). To establish the intraobserver reliability for measuring each parameter, two experienced orthopedic surgeons (M.M. and Y.Sa.) inputted all ROIs manually under 3D coordinates on this software, and then each parameter was measured automatically according to the software program. Grayscale images were segmented using a median filter to remove noise with a fixed threshold to extract mineralized bone components. We used a discriminant analysis method of image thresholding based on the density histogram of a selected ROI to ensure consistent image thresholding across all subjects studied. Isolated small particles in the marrow space and isolated small holes in bone were removed with a cluster-labeling algorithm to remove the small noise in the binary extraction. The measurement parameters calculated in 3D were the bone volume fraction, which indicates bone volume/total volume (BV/TV, %), trabecular thickness (Tb.Th, μm) (Fig. 2), trabecular separation (Tb.Sp, μm) (Fig. 2), and structure model index (SMI). The SMI is used to evaluate whether trabecular bone is rod-like or plate-like; a smaller value indicates a more plate-like structure [20, 21]. It has been established that good bone quality includes a higher BV/TV, higher Tb.Th, lower Tb.Sp, and lower SMI [13, 16].

Statistical analyses were performed using EZR software (Saitama Medical Center, Jichi Medical University, Saitama, Japan), which is the graphical user interface for R (The R Foundation for Statistical Computing, Vienna, Austria) [22]. More precisely, it is a modified version of R commander designed to add statistical functions frequently used in biostatistics. To minimize the effect of confounders, we standardized all data as follows: the discriminant analysis method was used to compare each ROI, all data were divided by the average for each individual as we used the optimal threshold value for each individual, and all data were standardized as the corrected ratio. The data are presented as mean ± standard deviation (SD). The trabecular microstructure parameters among ROIs were statistically evaluated by analysis of variance (ANOVA) and Tukey’s test. Inter- and intra-class correlation coefficients were used to assess inter- and intra-observer reliability. The significance level was set at *p* < 0.05.

## Results

In the apical area, the weight bearing part (superior site) had the highest bone microstructure quality, and bone quality in the central area was greater than in the inferior region. In the femoral neck, bone quality was greatest in the inferior region.

**Fig. 3:**
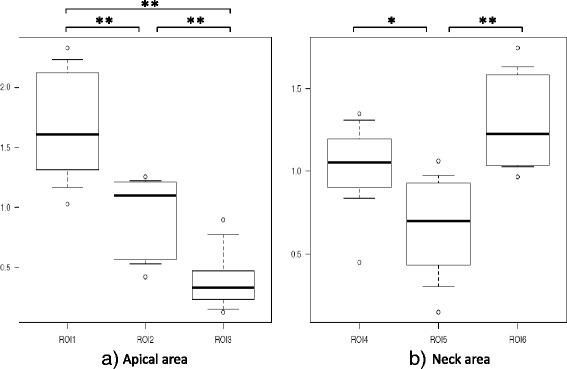
Bone volume/total volume (BV/TV) in the proximal head and neck. **a** The BV/TV in the apical area was significantly higher in ROI 1 than in ROI 2 and ROI 3, and was significantly higher in ROI 2 than in ROI 3. **b** The BV/TV in the neck area was significantly higher in ROI 6 than in ROI 5. *p < 0.05, **p < 0.01

BV/TV in the apical area of the femoral head was significantly higher in ROI 1 (1.65 ± 0.45) than in ROI 2 (0.95 ± 0.32) or ROI 3 (0.40 ± 0.25) (*p* < 0.01), and was significantly higher in ROI 2 than in ROI 3 (*p* < 0.01). In the neck, BV/TV was significantly higher in ROI 6 (1.30 ± 0.28) than in ROI 5 (0.67 ± 0.31) (*p* < 0.01), and significantly higher in ROI 4 (1.03 ± 0.26) than in ROI 5 (*p* < 0.05, Fig. 3). The intraobserver reliability was good, with values of 0.76. The interobserver reliability was also good at 0.74.

**Fig. 4:**
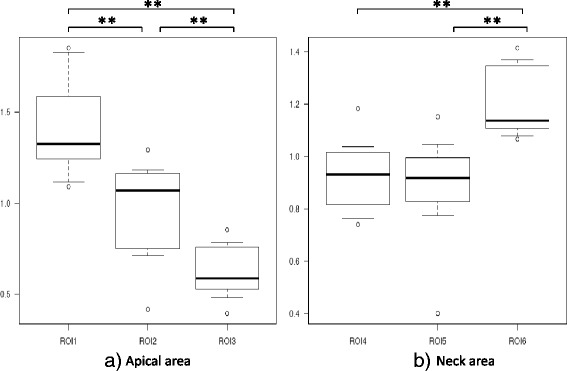
Trabecular thickness (Tb.Th) in the proximal head and neck. **a** The Tb.Th in the apical area was significantly higher in ROI 1 than in ROI 2 and ROI 3, and was significantly higher in ROI 2 than in ROI 3. **b** The Tb.Th in ROI 6 was significantly higher than in the other ROIs in the neck area. **p < 0.01

Tb.Th in the apical area was significantly higher in ROI 1 (1.40 ± 0.27) than in ROI 2 (0.98 ± 0.27) or ROI 3 (0.62 ± 0.14, *p* < 0.01), and was significantly higher in ROI 2 than in ROI 3 (*p* < 0.01). In the neck, Tb.Th was significantly higher in ROI 6 (1.19 ± 0.13) than in ROI 4 (0.92 ± 0.14) or ROI 5 (0.89 ± 0.20) (*p* < 0.01, Fig. 4).

**Fig. 5:**
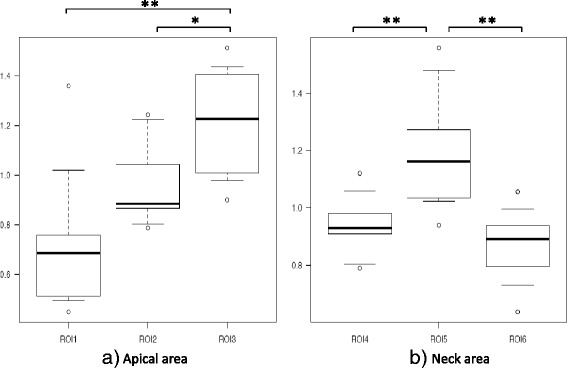
Trabecular separation (Tb.Sp) in the proximal head and neck. **a** The Tb.Sp in the apical area was significantly lower in ROI 1 than in ROI 2 and ROI 3, and was significantly lower in ROI 2 than in ROI 3. **b** The Tb.Sp in ROI 6 was significantly lower than in the other ROIs in the neck area. *p < 0.05, **p < 0.01

Tb.Sp in the apical area was significantly lower in ROI 1 (0.72 ± 0.27) than in ROI 3 (1.22 ± 0.22, *p* < 0.01), and was significantly lower in ROI 2 (0.95 ± 0.16) than in ROI 3 (*p* < 0.05). In the neck, Tb.Sp was significantly lower in ROI 4 (0.94 ± 0.10) than in ROI 5 (1.20 ± 0.20, *p* < 0.01), and was significantly lower in ROI 6 (0.87 ± 0.12) than in ROI 5 (*p* < 0.01, Fig. 5).

SMI in the apical area was significantly lower in ROI 1 (0.63 ± 0.09) than in ROI 2 (1.04 ± 0.17) or ROI 3 (1.32 ± 0.19, *p* < 0.01), and was significantly lower in ROI 2 than in ROI 3 (*p* < 0.01). In the neck, SMI was significantly lower in ROI 4 (0.97 ± 0.13) than in ROI 5 (1.13 ± 0.17, *p* < 0.05), and was significantly lower in ROI 6 (0.90 ± 0.11) than in ROI 5 (*p* < 0.01).

**Fig. 6:**
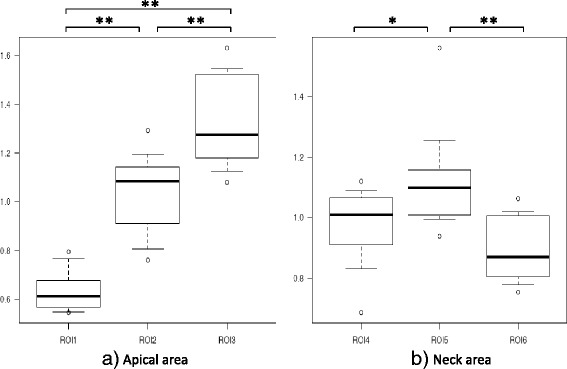
Structure model index (SMI) in the proximal head and neck. **a** The SMI in the apical area was significantly lower in ROI 1 than in ROI 2 and ROI 3, and was significantly lower in ROI 2 than in ROI 3. **b** The SMI in ROI 5 was significantly lower than in the other ROIs in the neck area. *p < 0.05, **p < 0.01

These results indicate that ROI 1 had the highest bone quality, and that ROI 2 had higher bone quality than ROI 3 in the apical area of the femoral head. In the femoral neck area, ROI 6 had the highest bone quality (Fig. 6).

## Discussion

The incidence of proximal femoral fractures caused by osteoporosis in Japan has increased each year. There were 148,000 proximal femoral fractures in Japan in 2007, which was 2.78 times higher than in 1987 [1]. Proximal femoral fractures cause elderly patients to become bedridden, decreasing their quality of life. Therefore, proximal femoral fractures are a major medical and social issue for the aging Japanese population [1, 23].

In Japan, it is recommended that elderly patients with proximal femoral fractures are treated surgically to ensure early rehabilitation and to help return the patient to their pre-injury status. We treat intertrochanteric femoral fractures with an intramedullary nail or compression hip screw. In both cases, precise reduction and appropriate lag screw placement lead to good clinical results. Lag screw cut-out is a serious complication of intertrochanteric fracture repair, with an incidence of 1–7 % [4, 24–27]. The most important predictive factor for lag screw cut-out is tip–apex distance, which is the combination of AP and lateral distances from the tip of the screw to the apex of the femoral head [28], followed by screw position, fracture pattern, reduction, and patient age [4]. The tip–apex distance is closely associated with lag screw positioning. Therefore, optimal placement of the lag screw is very important to reduce complications. Placement of the screw in the central or inferior region of the femoral head as seen on the AP view and in the central region as seen on the lateral view is considered as optimal positioning [29]. These recommendations are based on empirical radiographic evaluation, but few studies have evaluated the importance of bone quality for optimal screw positioning.

In this study, we evaluated bone quality in the femoral head by measuring trabecular microstructure using in vivo MDCT images. In the apical area of the femoral head, bone quality was highest in the superior region (ROI 1). The superior region is the location of the principal compressive group of trabeculae and is a weight-bearing area; therefore, bone quality parameters are high. However, radiographic investigation found that the rate of lag screw cut-out in the superior area was higher than in other regions [4, 28, 30]. In a biomechanical study, the insertion of lag screws in the superior region of the femoral head reduced the fixing force, possibly resulting in poor outcomes [31–33]. Based on clinical results and biomechanical analysis, placing the lag screw in the superior region of the femoral head is not recommended [3, 5–10, 32, 33]. In our study, bone quality in the central area was greater than in the inferior area in the femoral head apex. In the femoral neck, bone strength was highest in the inferior area where the principal compressive group of trabeculae is located. Jenkins et al. reported that the center of the femoral head has the highest bone strength on the equatorial plane based on measurement of trabecular microstructure with μCT in removed femoral heads [11]. They recommended that lag screws should be placed in the center of the femoral head to achieve optimal fixation. In contrast, biomechanical studies have found that the most stable position for lag screw placement is the inferior region of the femoral head [32, 33]. Different conclusions are reached based on bone quality versus biomechanics; an understanding of both factors is important to achieve excellent clinical results.

This study had some limitations. First, it has a relatively small sample size. However, despite the small sample size, we consider our data to be important as we found a significant statistical difference in bone quality of the femoral head for each microstructure parameter. Further study evaluating a healthy control group and age-specific bone quality is warranted. Second, we did not evaluate whether inserting the lag screw in an area of good bone quality prevented cut-out. Previous studies on other bone parts have shown a correlation between bone quality and fixation strength of prosthesis for fractures [34, 35]. We consider that bone quality of the local area is one of the most important aspects for attaining strong fixation. Last, we have not evaluated the accuracy of MDCT data; however, Baum et al. reported that there is no difference between MDCT data and high resolution peripheral quantitative computed tomography data regarding trabecular microstructure and bone quality in vertebral bone [36], hence we consider that MDCT is also a useful tool to evaluate trabecular microstructure of the femoral head.

## Conclusions

In conclusion, MDCT in vivo was a feasible technique to assess bone quality in patients with intertrochanteric fracture. In cases of intertrochanteric fracture, the best location for the lag screw is still controversial; the best location should be determined by using all information available from the empirical surgery outcome, biomechanical results, and bone quality obtained by MDCT.

## Abbreviations

AP: anteroposterior
BV/TV: bone volume/total volume
μCT: micro computed tomography
MDCT: multidetector row computed tomography
ROI: region of interest
SMI: structure model index
Tb.Th: trabecular thickness
Tb.Sp: trabecular separation
